# A Practical Guide for Harnessing Phylogenomics in Biocontrol: Accounting for Topological Uncertainty and Phylogenetic Distance in the Centrifugal Phylogenetic Method and Beyond

**DOI:** 10.1111/eva.70203

**Published:** 2026-02-24

**Authors:** Stephanie H. Chen, Michelle Rafter, Ben Gooden, Alexander N. Schmidt‐Lebuhn

**Affiliations:** ^1^ CSIRO, Centre for Australian National Biodiversity Research (a Joint Venture Between Parks Australia and CSIRO) Canberra Australian Capital Territory Australia; ^2^ CSIRO Health and Biosecurity Brisbane Queensland Australia; ^3^ CSIRO Health and Biosecurity Canberra Australian Capital Territory Australia

**Keywords:** degrees of separation, *Passiflora*, patristic distance, phylogenomics, Senecioneae, weed biological control

## Abstract

In the genomic era, phylogenomics is playing an increasingly important role in biological control research for prioritising species in host specificity testing, species delimitation, and elucidating the origins of introduced species. This paper outlines key concepts in phylogenomics relevant to biocontrol practitioners and provides practical guidance on the construction and interpretation of phylogenetic trees. We examine the patterns and distributions of degrees of separation and phylogenetic distance (also known as patristic distance) across different types of phylogenetic trees, including cladograms, phylograms, and chronograms, and offer recommendations for their application. Further, we consider the impact of topological uncertainty on these distance measures and the inferences they inform for decision‐making in biological control. These concepts are illustrated through two case study datasets representing distinct evolutionary contexts. The first explores a recently published phylogeny of Asteraceae tribe Senecioneae derived from traditionally used nuclear and chloroplast Sanger molecular markers, using common groundsel ( *Senecio vulgaris* ) as the hypothetical target weed. The second case study dataset focuses on the biocontrol of stinking passionflower ( *Passiflora foetida* ) in Australia, presenting a novel target capture (Angiosperms353) phylogeny for this group. Equipping biocontrol practitioners with a deeper understanding of phylogenomics will facilitate more efficient and data‐driven decision‐making in biological control.

## 1 Introduction

The democratisation of DNA sequencing has unlocked the potential to leverage the increasing volumes of sequencing data available in public databases for applications in fields such as biological control. With advances in sequencing and phylogenomic methods and their decreasing cost, it is becoming increasingly feasible to generate datasets to answer specific questions for biocontrol (Gaskin et al. 2011; Gaskin 2024). This includes using molecular datasets to prioritise species for host specificity testing (Chen et al. 2024) and studying the geographic origin of species and species delimitation (Scott et al. 1998; Milne and Abbott 2004; Gildenhuys et al. 2015; Chen, Grealy, et al. 2025).

In the present contribution aimed at practitioners of biological control, we discuss a genomics era approach to harness phylogenomics to inform host specificity testing in weed biological control. While phylogenetics has long been used to infer evolutionary relationships among species, its application in biocontrol remains somewhat limited and often oversimplified. We argue that recent advances in phylogenomics offer underutilised opportunities to refine risk assessments. To guide biocontrol researchers in applying these tools more effectively, we emphasise three key recommendations: (1) incorporate branch lengths and divergence times rather than relying solely on node counts to assess relatedness; (2) explicitly account for topological uncertainty when interpreting phylogenetic trees; and (3) invest in generating better phylogenetic data or build collaborations with phylogeneticists and systematic botanists.

We cover the basics of how to read phylogenetic trees, including interpreting branch lengths and ways of accounting for phylogenetic uncertainty when calculating phylogenetic distance measures in the context of biological control. We provide an overview of generating and visualising a phylogeny and offer recommendations on the computational tools to use. Finally, we use two datasets that showcase different evolutionary histories and marker systems (Sanger sequencing vs target capture) to illustrate the effect of phylogenetic uncertainty on degrees of separation and phylogenetic distance. The first dataset is a recently published phylogeny of the Asteraceae tribe Senecioneae from four traditionally used Sanger markers, and the second is a new *Passiflora* phylogeny from target capture sequencing (TCS) using the Angiosperms353 bait set in the context of stinking passionflower biocontrol in Australia.

## 2 Anatomy of a Phylogenetic Tree

**FIGURE 1:**
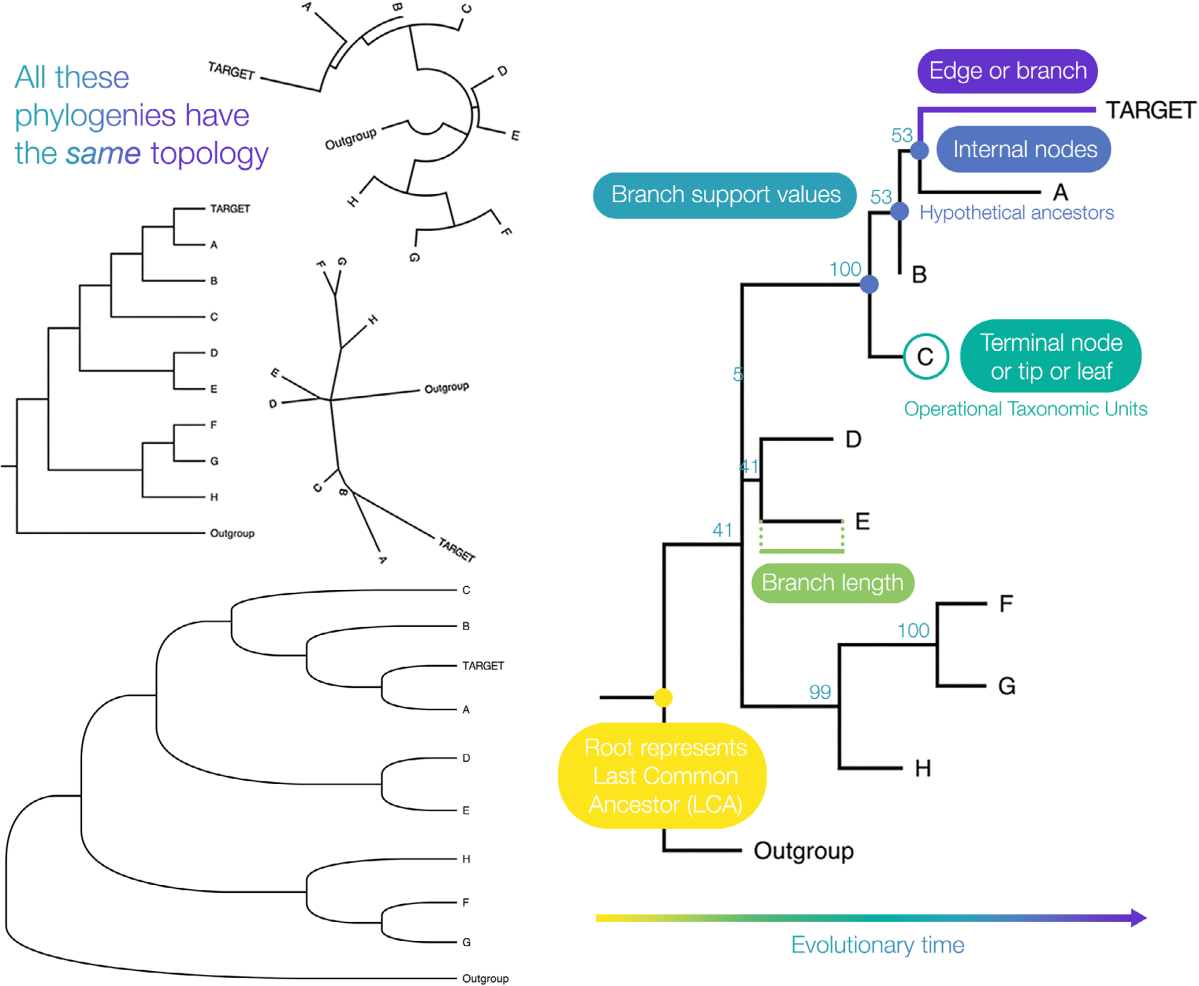
Different ways of visualising one tree topology and parts of a bifurcating phylogenetic tree with different species at each terminal node. Note that the phylograms contain a 0 length branch where the clade containing A, B, and C splits off, and there are no polytomies.

A phylogenetic tree represents evolutionary relationships between organisms (species trees—the focus of this paper), specimens/samples, or gene sequences (gene trees). The most important aspect of a phylogenetic tree is its topology or branching order, which is the arrangement of nodes relative to each other. However, trees with identical topology can be visualised in dissimilar, potentially confusing ways, or with different kinds of branch lengths (Figure 1). Phylogenetic trees may be visualised and annotated using tools such as FigTree (Andrew Rambaut 2018) and the R package ggtree (Yu et al. 2017).

Identical topology can have very different implications for evolution depending on how a phylogenetic tree is rooted (Kinene et al. 2016). Most phylogenetic approaches are ‘time‐reversible’ and produce unrooted trees, which then must be rooted afterwards to provide them with directionality. The most frequently used method is outgroup rooting, which involves polarising the tree on one or more species that the researcher is confident are outside of the study group but relatively closely related to it.

## 3 The Centrifugal Phylogenetic Method and Distance Measures in Biological Control

**FIGURE 2:**
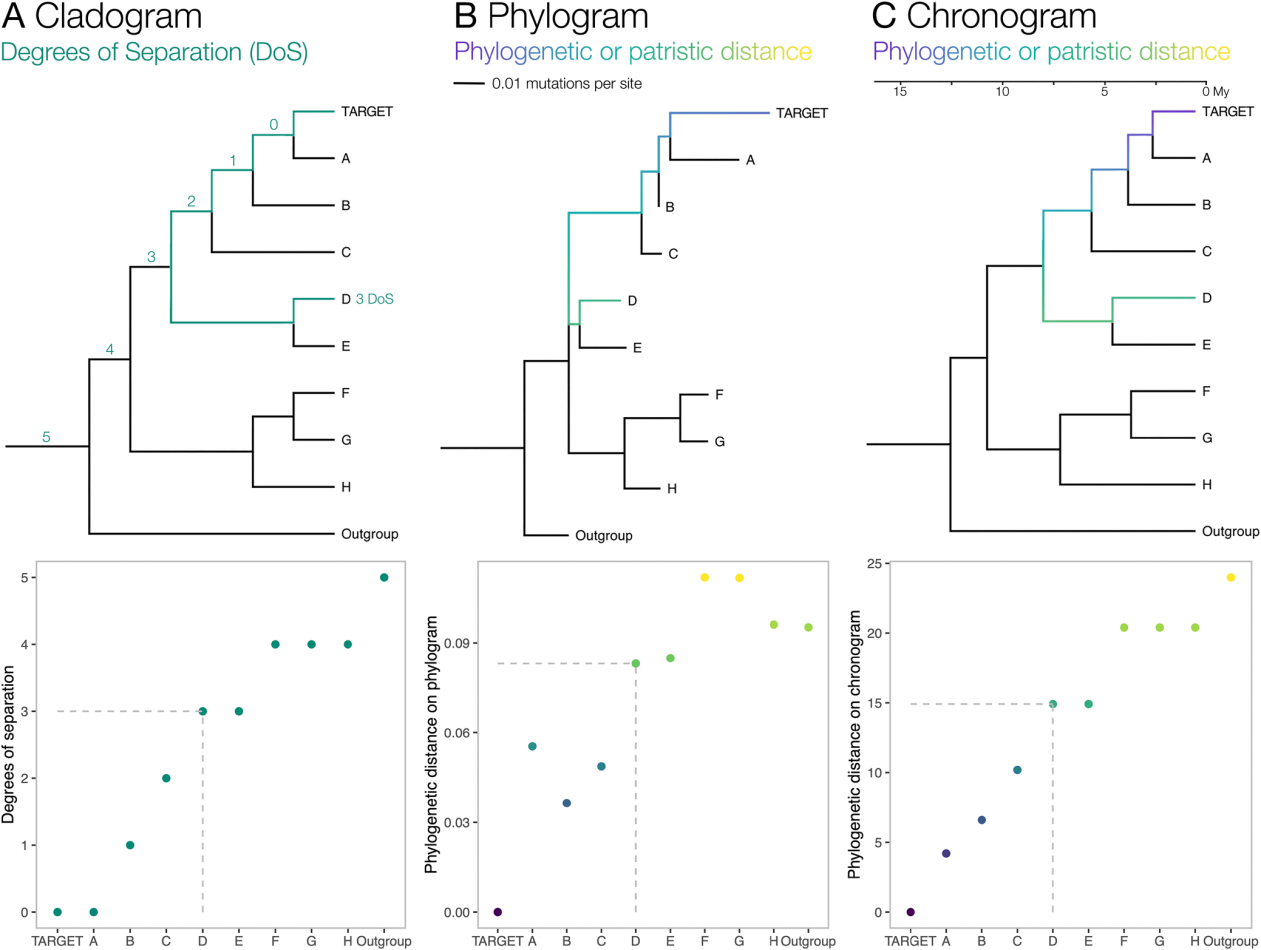
Illustration of three measures of relatedness on a phylogenetic tree between an example biocontrol target species and species D. (A) Degrees of separation, counting the lineage splits between the target and its common ancestor with D. (B) Phylogenetic (patristic) distance on a phylogram, indicating the estimated changes per character separating the two species. Root‐tip path lengths vary strongly because of differences in the estimated rates of evolution. (C) Phylogenetic distance on a chronogram, which is equal to two times the estimated time since divergence of the lineages leading up to the two species. Data taken from a portion of Senecioneae phylogeny used in the Case Study.

The centrifugal phylogenetic method is widely used for assembling host specificity test lists (Wapshere 1974; Briese 2002, 2006; Gilbert et al. 2012). It ensures that the candidate agent is tested against plant species with different degrees of relatedness to the target weed. Originally, taxonomic ranks were used to guide the assembly of test lists, for example, by picking representative plants from the same genus as the target, the same tribe, the same subfamily, the same family, etc. (Wapshere 1974). As published phylogenies have become available for an increasing number of plant groups, their topologies have been used directly to infer relatedness between the target and other species (Kelch and McClay 2004), reflecting the observation that the likelihood of two plants sharing the same herbivore or pathogen decreases with increasing phylogenetic distance (Gilbert and Webb 2007). The most frequently used measure of relatedness is degrees of (phylogenetic) separation, simply a count of the number of lineage splits between the target species and its common ancestor with a potential test plant (Briese and Walker 2002; D. T. Briese 2005; Taylor and Dhileepan 2019). The sister clade of a target species is scored as having zero degrees of phylogenetic separation, the next most recent common ancestor is scored as one, and so on further down the phylogeny (Figure 2A).

This approach has, however, two main disadvantages. First, counting of lineage splits (i.e., node counting) does not make use of the information provided by the branch lengths of phylogenetic trees. An approach that accounts for branch length information would show natural breaks in the order of relatedness for cases such as when degrees of separation of 1, 2, and 3 are all very close to the target but 4 is much further away. An alternative measure of relatedness would be the phylogenetic (or patristic) distance between the target and another species in a phylogenetic tree, that is, the sum of the length of the tree branches separating the two (Figure 2B,C).

Second, counting ancestral nodes on a given phylogenetic tree does not account for phylogenetic uncertainty. Many phylogenies contain hard‐to‐resolve relationships, indicated by internal branches being very short and/or showing low branch support (Bayesian posterior probability < 0.95 and/or bootstrap < 70%) (Figure 2B,C). Degrees of separation may be misleading if the true phylogeny differs from the inferred one in one or two crucial tree nodes because each branching event counts equally regardless of our confidence in its position.

The behaviour of measures of relatedness can be considered across increasing phylogenetic distances. Phylogenetic distances on a chronogram increase monotonously with increasing degrees of separation, but not at constant intervals (Figure 2C). This means that they may show natural breaks in relatedness. In cases where a lineage is isolated due, for example, to the extinction of all its closer relatives, this fact will be reflected by a jump in the phylogenetic distance score. On the other hand, on a chronogram, the rank order of phylogenetic distances from a target will always be identical to that of the degrees of separation. Using degrees of separation may result in a different ranking of species compared to patristic distance.

On a phylogram, the phylogenetic distance from the target can sometimes be lower for a species with a higher degree of separation, reflecting the variation of rates of evolution in different parts of the phylogeny (Figure 2B). Whereas on the chronogram all species of a clade not including the target have a constant phylogenetic distance from the target (Figure 2C), on a phylogram this distance varies for each member of a clade.

## 4 Interpreting Branch Lengths of Phylogenetic Trees

### 4.1 Cladograms: Undefined or No Branch Lengths

Phylogenetic trees that only indicate the inferred branching order but do not have defined branch lengths are called cladograms. They are relatively rare in recent literature but are still found in the form of consensus trees or supertrees summarising topology information from other trees.

The calculation of phylogenetic distances on cladograms (Figure 2A) is equivalent to node counting, as there is a lack of branch length information, so that all branches can only be treated as equal‐length. Such arbitrarily equal branch lengths do not reflect any biological reality in terms of genetic, ecological, or physiological differentiation. The path length between two terminals would be determined entirely by two factors: sampling completeness and phylogenetic isolation. A very isolated lineage may be scored as phylogenetically close to another species despite it being distantly related. Additionally, species nested deeply within a clade of many very close relatives would have an inflated mean phylogenetic distance from other tree terminals, resulting in scores that subvert the purpose of the concept of phylogenetic distance (Elliott et al. 2018). Degrees of separation are partly robust to this problem by counting all members of a non‐target clade as equidistant from the target, but this problem still applies to some degree along the ancestral lineage of the target.

### 4.2 Phylograms: Branch Lengths Indicate Character Changes

Phylograms are produced by most analyses that do not use a clock or coalescent model, and with widely used phylogenetic software such as MrBayes, RAxML, PhyML, IQ‐TREE, or PAUP. The branch lengths on phylograms indicate either the inferred absolute number of character changes (parsimony analysis) or the estimated number of changes per character (likelihood and Bayesian analyses), so that the terminals of the tree are at varying distances from the root.

The main conceptual problem of using phylograms for the calculation of phylogenetic distances (Figure 2B) is that the character changes shown as branch lengths are those in the data used for phylogenetic analyses, which, despite the increasing availability of genomic scale data, are currently often non‐coding ribosomal or chloroplast spacer regions as opposed to protein‐coding regions whose character changes are likely to affect phenotypes that influence the host specificity of pathogens and pests. Some researchers argue that the rate of evolution in non‐coding regions is likely to be correlated with the rate in evolutionarily or ecologically relevant genes, as both would be influenced by generation time and rate of metabolism (Gillooly et al. 2005; Rosauer et al. 2009; Faith et al. 2009; Lanfear et al. 2013), but others doubt that this correlation has been demonstrated (Mooers and Redding 2009). This is relevant where, for example, a clade of four species at a degree of separation two contains one species that ‘sticks out’ with a higher rate of evolution than the three others. A phylogenetic distance matrix calculated from a phylogram would show it as considerably more distant from the target species than the other three members of the clade, and perhaps even more distant than species at higher degrees of separation, but it would be unclear if that is ecologically meaningful.

### 4.3 Chronograms: Branch Lengths Indicate Time Since Divergence

Chronograms are produced by likelihood or Bayesian analyses under a clock or coalescent model, for example, by software such as BEAST and MrBayes or through time‐calibration of pre‐existing phylograms or cladograms with software such as r8s (Sanderson 2003), treePL (Smith and O'Meara 2012), or MCMCtree (Yang 2007). The branch lengths on chronograms indicate the estimated time between two lineage splits, generally in millions of years, so that extant terminals of the tree are necessarily equidistant from the root.

It can be argued that chronograms quantify the relatedness of species in the sense with which this term is generally understood: out of three organisms, those two that are most closely related share the most recent common ancestor (Figure 2C). Conversely, branch length information on chronograms does not consider that a lineage may have undergone much more rapid change than its sister lineage. To correlate phylogenetic distance with ecological and physiological similarity one must assume clock‐like evolution, or at least the absence of major shifts in the rate of evolution.

## 5 Accounting for Topological Uncertainty

Phylogenetic trees always remain hypotheses based on the best available data and shaped by methodological assumptions. For any meaningfully large dataset, it is impossible to be certain that the ‘best’ phylogenetic tree has been found, because the search space of possible phylogenetic trees is extremely large—for a set of 20 samples, there are ca. 221 trillion possible unrooted tree topologies. This inherent uncertainty in tree topology must be accounted for, as incorrect or poorly supported topologies can lead to misleading inferences about evolutionary relationships. In the context of biocontrol, these errors may result in inappropriate prioritisation of species for host specificity testing.

Topological uncertainty directly affects measures of relatedness which are used to infer ecological and chemical similarity which relates to the potential for non‐target effects. There are two main ways of accounting for topological uncertainty. In the degrees of separation framework (or any approach based on node counting) poorly supported nodes may be collapsed onto the next more basal node, creating (soft) polytomies (Maddison 1989). This practice reduces the degrees of separation further down the phylogeny and treats all clades that are potentially of a given degree at that degree. The use of polytomies is, however, not feasible for phylogenetic distances calculated from branch lengths, as artificially created polytomies would not have well‐defined branch lengths. Alternatively, phylogenetic uncertainty may be quantified for any measure of relatedness by sampling across near‐optimal tree space. A sample of trees from the posterior distribution of a Bayesian analysis or bootstrap trees could be used to calculate a mean and standard deviation for the relevant measure, providing a more robust basis for downstream decisions.

Missing data impacts contribute to uncertainty in measures of relatedness. Unsampled species or incomplete sequence data are common due to logistical constraints, limited taxonomic knowledge, or lack of molecular resources. These gaps tend to affect node‐counting approaches (degrees of separation) more severely than methods that are based on branch length (phylogenetic distance). Additionally, reconciling multiple published phylogenies introduces uncertainty, especially when the trees are based on different underlying datasets and methods. Discordance between phylogenies inferred from chloroplast and nuclear genomes for the same set of samples is well documented (Soltis and Kuzoff 1995) and can complicate assessments of relatedness.

Measures of relatedness and topological uncertainty need to be considered when interpreting phylogenetic trees. Error bars are typically larger for degrees of separation and phylogenetic distance on a chronogram than for phylogenetic distance on a phylogram. This is presumably because topological uncertainty has larger effects on phylogenetic distances if branch lengths are not correlated with rates of change. In all cases, careful consideration of topological uncertainty is essential for interpreting relatedness and ensuring that phylogenetic insights are translated into reliable biocontrol risk assessments.

## 6 Building Your Own Phylogenetic Tree

If you are moving beyond interpreting published phylogenies from the literature to constructing your own, whether using publicly available sequencing data or data from a new sequencing experiment, getting started in phylogenomics can be an intimidating endeavour. Numerous decisions must be made about what molecular markers to use, how to sample, which bioinformatic and phylogenetic software packages to use, and how to parameterise each tool, so you may find yourself ‘stuck in the weeds’.

Many excellent guides already exist for getting started in phylogenomics (e.g., Cvrčková 2016; Kapli et al. 2020; Lozano‐Fernandez 2022). Here, we provide practical advice tailored to biocontrol practitioners working on invasive plants. We focus on the phylogenetic analysis step of generating the tree, rather than the prior steps of processing the raw data. We provide a non‐exhaustive selection of recommended software for phylogenomic analyses depending on the type of sequencing data obtained and discuss considerations such as computational requirements and data reusability.

**FIGURE 3:**
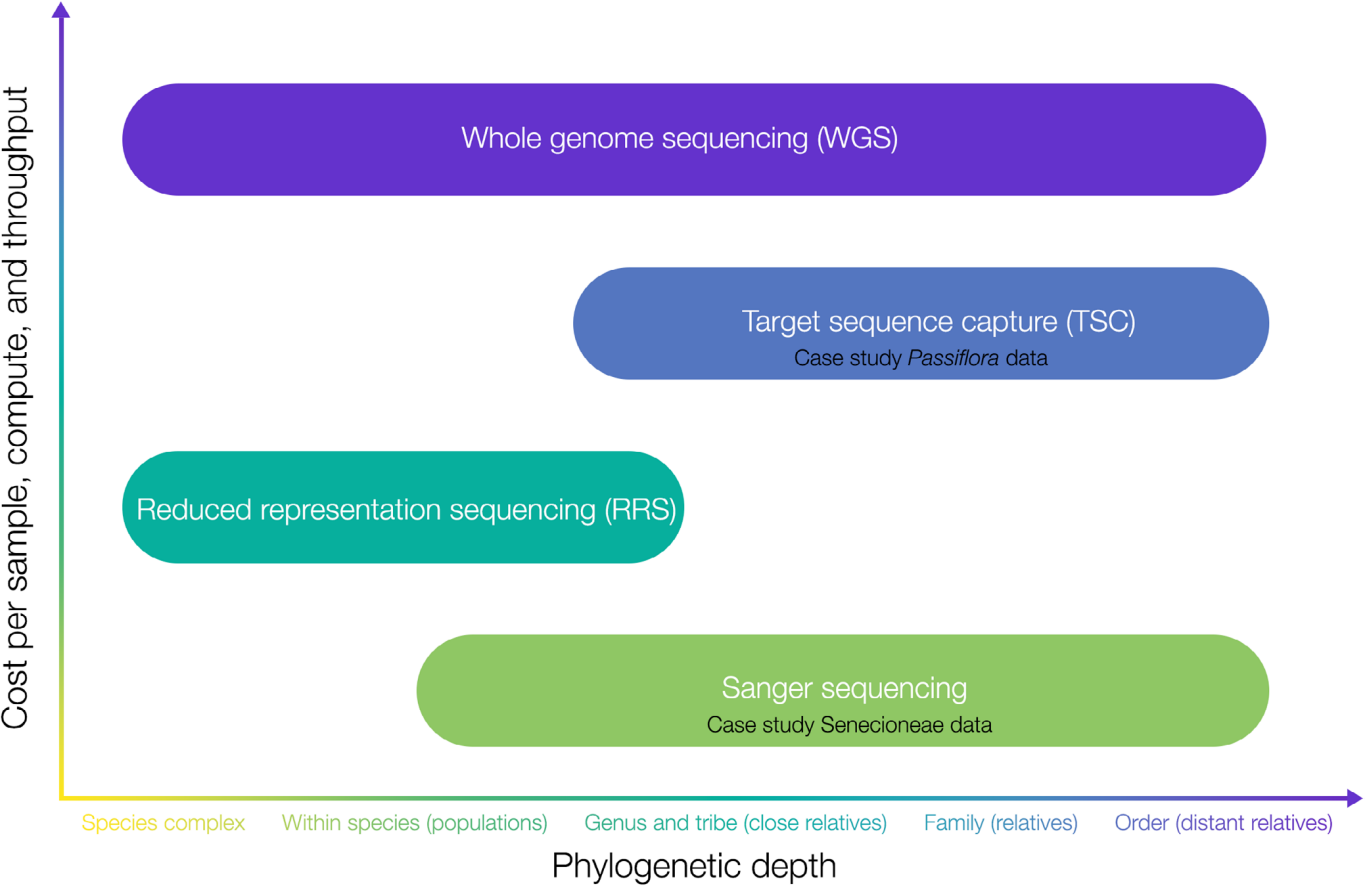
Phylogenetic depth vs. cost per sample and computational resources required for different types of sequencing for phylogenomics in weed biological control. Note that while cost per sample increases with throughput, the cost per base pair typically decreases with increasing throughput.

As increasing proportions of the genome are sequenced with high throughput methods such as target capture sequencing (TCS) and whole genome sequencing (WGS), the cost of sequencing and computational resources and bioinformatics expertise needed increases (Figure 3). Sequences from Sanger sequencing, which target specific well‐studied loci and can include both nuclear and chloroplast markers (see Senecioneae example in Case Study), can be readily processed on a local computer. For example, a study using the two markers *ITS1* and *trnL‐F* and Bayesian analysis with MrBayes within the Anacardiaceae informed the biocontrol of *Schinus terebinthifolia* (Brazilian peppertree) (Wheeler and Madeira 2017). Another study using the *trnL* and *trnL‐F* regions aimed to determine the relationship of *Lygodium microphyllum* to other *Lygodium* species (Madeira et al. 2008). However, Sanger sequences, characteristic of older studies, can result in poor phylogenetic resolution such as when four markers (*trn*H‐*psb*A, matK, *trn*T‐*trn*L, and GBSSI) were used to investigate African boxthorn ( *Lycium ferocissimum* ) and relatives using Bayesian phylogenetic methods (McCulloch et al. 2020).

Two widely used types of reduced representation sequencing (RRS) are DArTseq (Diversity Arrays Technology) and RADseq (restriction‐site associated). These methods use restriction enzymes to generate sequence tags that contain single nucleotide polymorphisms (SNPs) and invariant sites, and rely on short‐read sequencing platforms. SNP data from RRS may be processed on a local computer, but if the sequencing centre provides only raw read data, more compute power (i.e., High Performance Computing or HPC) may be required to process the data into filtered SNPs ready for analysis. The analysis of large volumes of DNA sequence data, such as produced by TCS (see *Passiflora* example in Case Study), and WGS requires high performance computing (HPC).

Data reusability varies across sequencing technologies. Sanger markers are highly reusable, but the field is shifting to more advanced methods to mitigate the issue that small numbers of markers may not have sufficient power for resolution. RRS has low data reusability due to the specificity of restriction enzymes and optimisation needed for each sequencing experiment; SNP data from one experiment are not homologous to those from another, even in a closely related plant group. Target capture sequencing can be reused across experiments, but only within the group that the markers were designed for. The *Plant and Fungal Trees of Life (PAFTOL*) project (Baker et al. 2022) has created resources such as reference sequences across all flowering plants and the Angiosperms353 bait kit (353 nuclear genes), which can be readily leveraged in your own studies for weed biological control. WGS has high reusability, although a reference genome is needed for some types of downstream analysis.

Budget will be a determining factor of the type of sequencing and number of samples. When planning a sequencing experiment, it is recommended to check repositories such as GenBank for already published data to avoid duplicated efforts, taking into consideration the source and reliable identification of specimens if you intend to incorporate these sequences into your analysis. Outsourcing the DNA extraction, library preparation, and sequencing removes the need for in‐house technical expertise and equipment in these areas.

Herbarium specimens work well with target‐capture. On the other hand, methods such as RNA‐seq for sequencing transcriptomes only work reliably with freshly sampled plant tissue, so they are not as useful for application in biocontrol, especially if rare or remotely occurring species must be sampled.

Sequencing centres can also assist with the design of an experiment and provide some troubleshooting, but more importantly, the desired end goals and outputs must be clear. Is the goal to obtain a well resolved and comprehensively sampled species tree for your biocontrol target for risk assessment? Are there additional questions surrounding the target and relatives that can be answered with genomic data, for example, regarding hybridisation, species delimitation, establishment means, or genetic diversity? RRS and SNP data are more suited to these shallow phylogenetics questions (population and species complex level, and weeds and their close relatives), whereas target capture sequencing is most appropriate for deeper phylogenetics (highly diverged species) (Figure 3). For example, a seven nuclear marker (ETS, ITS, and five PPR loci) phylogeny of *Lantana* and allied genera, including ornamentals and invasives, demonstrated the non‐monophyly of *Lantana* and *Lippia* (Lu‐Irving et al. 2021), shedding light on previous non‐target attacks (Hinz et al. 2019). A subsequent study at the population and species complex level used over 10,800 SNPs from DArTseq to characterise morphotype lineages in *Lantana camara* to inform agent selection (Lu‐Irving et al. 2022). Another study on *Chrysanthemoides monilifera* ssp. *rotundata* (bitou bush) obtained 20,221 SNPs from ddRADseq; phylogenetic analysis was used to determine the origin of introduced populations, and the genetic diversity and structure of populations was also investigated (Byrne et al. 2022).

The choice of phylogenetic method (Table 1) will largely depend on the volume and type of sequencing data available and the computational resources at your disposal. The required compute will be determined by the type of sequencing data as well as the number of samples. For three taxa, there is only one possible phylogenetic tree. For 10 taxa, there are over 34 million possible bifurcating trees—the tree space to search vastly increases (Felsenstein 1978).

**TABLE 1:** An overview of phylogenetic methods for weed biological control.

Phylogenetic method	Example software	How it works	Speed and recommended use case	Output phylogeny
Distance and clustering	PAUP (Wilgenbusch and Swofford 2003), MEGA (Tamura et al. 2013)	Clusters samples based on a matrix of pairwise distances.	Fast, but not considered state‐of‐the‐art for phylogenetic analysis except for data exploration.	Neighbour Joining produces phylograms, UPGMA produces ultrametric trees (chronogram‐like but not time‐calibrated).
Parsimony	PAUP (Wilgenbusch and Swofford 2003), TNT (Goloboff et al. 2008), MEGA (Tamura et al. 2013)	Searches for the phylogeny that implies the lowest required number of trait changes to explain a single data matrix.	Not considered state‐of‐the‐art by most evolutionary biologists and today mostly used for morphological data, for example, in palaeontology.	Phylograms where branch lengths indicate the inferred number of character changes.
Maximum likelihood	PAUP (Wilgenbusch and Swofford 2003), MEGA (Tamura et al. 2013), PHYLIP (Felsenstein 1993), RAxML (Stamatakis 2006), IQ‐TREE (Minh et al. 2020)	Searches for the phylogeny that explains a single data matrix with the highest likelihood given an assumed model of evolution (i.e., of nucleotide substitution in DNA sequence data).	Currently one of the preferred approaches for datasets too large for Bayesian analysis.	Phylograms where branch lengths indicate estimated changes per character.
Bayesian phylogenetics	MrBayes (Huelsenbeck and Ronquist 2001), RevBayes (Höhna et al. 2016), BEAST (Drummond and Rambaut 2007)	Samples tree space around the phylogeny that explains a single data matrix with the highest likelihood given an assumed model of evolution (i.e., of nucleotide substitution in DNA sequence data) and returns a posterior probability distribution instead of a single ‘best’ phylogeny.	Currently state‐of‐the‐art in evolutionary biology where datasets are small enough for Bayesian phylogenetics to be computationally feasible.	BEAST only implements clock models and therefore always produces ultrametric trees. MrBayes and RevBayes can produce phylograms or ultrametric trees.
Bayesian species tree estimation	StarBEAST (Ogilvie et al. 2017)	Samples tree space around the species phylogeny that explains a dataset of multiple gene alignments that may contain multiple samples from some species with the highest likelihood.	Currently state‐of‐the‐art in evolutionary biology where datasets are small enough for Bayesian phylogenetics to be computationally feasible.	StarBEAST only implements clock models and therefore always produces ultrametric trees.
Two‐step coalescent	ASTRAL suite (Zhang et al. 2018, 2020)	Infers the most likely species phylogeny based on multiple gene phylogenies that may contain multiple samples from some species.	Fast, even for very large datasets, but has the weakness of assuming that gene tree topologies are well resolved and supported.	Internal branch lengths are given in coalescent units and terminal branch lengths may be undefined. Programs such as CASTLES (Tabatabaee et al. 2023) can be used to convert branch lengths to substitutions per site unit.

For Sanger sequences, we recommend concatenation with maximum likelihood methods using IQ‐TREE (Minh et al. 2020), or Bayesian inference. While plastid and nuclear ribosomal markers are routinely concatenated for phylogenetic analysis, there may be incongruence between plastid and nuclear phylogenies, so these are best analysed separately. For RRS, quartet‐based methods can be used for phylogenies, for example, SVDquartets (Chifman and Kubatko 2014), but phylogenetic network methods such as Splitstree (Huson 1998) can be more insightful where gene flow is being investigated. For TCS and WGS, concatenation with maximum likelihood methods using IQ‐TREE and/or coalescent methods using ASTRAL and its variants (Zhang et al. 2018, 2020; Zhang and Mirarab 2022) are recommended for phylogenomic analysis. In general, TCS will produce better resolved trees with less phylogenetic uncertainty than studies using Sanger sequencing.

To make the most of WGS data, other analyses such as comparative and functional genomics are needed. Additionally, WGS can be applied with different sequencing technologies, that is, short and long reads, and this impacts the downstream inferences that can be made. WGS data can be particularly valuable for species with complex evolutionary histories and complex genomes. For example, many sequencing efforts have been directed towards *Phragmites australis* (common reed) to support management, as it is a damaging and widespread weed across the United States and has a complex history of introductions, and the species comprises many lineages (Lindsay et al. 2023). A PacBio reference genome with 64,857 annotated protein‐coding gene models was assembled along with transcriptomes (Illumina RNA‐seq), and analyses revealed differences between invasive and native genotypes (Oh et al. 2022). These genomic resources enhance our understanding of invasiveness in *Phragmites* and other grass species, and support the development of more effective management strategies by identifying genetic targets for biocontrol. Another example is the genome assembly of horseweed ( *Erigeron canadensis* ) alongside Illumina WGS of seven horseweed biotypes to investigate herbicide resistance and develop new management strategies (Peng et al. 2014). However, WGS is currently still rarely used for understanding targets and relatives in weed biocontrol.

Building a robust phylogenetic tree is inherently an iterative process that involves quality control and validation, such as investigating outliers to curate the tree. There is no universally optimal method, so a valuable strategy is to take multiple approaches and compare the outputs. For example, with target sequence capture data, it is common to perform a concatenated analysis using maximum likelihood as well as a two‐step coalescent analysis (Chen et al. 2024). Additionally, the phylogenies produced will typically be time‐reversible (unless inferred under clock models, e.g., with BEAST), so a further step of time calibration is needed for producing a chronogram (Forest 2009). For large phylogenies, penalised likelihood (Sanderson 2002) is a commonly used method and can be implemented through r8s software (Sanderson 2003) or via the chronos function in the R package ape (Paradis et al. 2004). Another program commonly used for time calibration is MCMCtree which is available through PAML (Yang 2007), but may also be implemented through the R package MCMCtreeR (Puttick 2019).

It is also important to be aware of errors during tree building; stochastic errors arise from short sequences (insufficient data) whilst systematic errors occur when the model assumptions are violated (Philippe and Telford 2006). The prevalence of random errors is decreasing in the genomic era due to the increasing amounts of sequencing data being used. However, systematic errors are increasing with the use of longer alignments (Yu et al. 2017). As the cost of sequencing continues to decrease with technological advances, we expect a trend towards whole genome sequencing, as well as a transition from short‐read to long‐read sequencing. This will allow for phylogenomic inferences from more of the genome.

## 7 Case Study: Phylogenetic Distance Measures and Uncertainty in a Sanger Dataset of Senecioneae Versus a Target Capture Sequencing Dataset of *Passiflora*

To examine the behaviour of degrees of separation and phylogenetic distance across increasing distance from the target species and when accounting for topological uncertainty, we compared data from recently published Sanger phylogenies of the Senecioneae (groundsel tribe, Asteraceae) (Schmidt‐Lebuhn et al. 2020) to a target sequence capture phylogeny of *Passiflora* (Passifloraceae). The original purpose of generating both datasets was to inform the design of weed biocontrol test lists.

The Senecioneae phylogeny was inferred from a supermatrix of four gene regions—nuclear ribosomal ETS and ITS and chloroplast *psb*A‐*trn*H and *trn*L‐*trn*F sequences—using maximum likelihood analysis in IQ‐TREE v1.4.4 (Minh et al. 2020). The alignment contained 4092 columns with 1312 parsimony‐informative sites. The phylogeny contained 1154 tips. The resulting phylogram was time calibrated with penalised likelihood (Sanderson 2002) implemented in the chronos function of the R package APE (Paradis et al. 2004). *Doronicum* species were used to outgroup‐root the tree. We used two secondary calibration points: 25–46 Mya for the most recent common ancestor of Senecioneae including *Abrotanella*, and 39–48 Mya for the split between *Doronicum* and the Senecioneae, the root of the tree (Panero and Crozier 2016). Testing the strict, correlated, relaxed and discrete clock models showed correlated to be favoured. For this case study, we selected common groundsel ( *Senecio vulgaris* ), a widespread weed, as our hypothetical target for the calculation of measures of relatedness.

Stinking passionflower ( *Passiflora foetida* ) is a target of biological weed control in Australia, and a host test list was previously developed (Kumaran et al. 2020). *Passiflora* is phylogenetically isolated in Australia, with five native species, and this has been confirmed using chloroplast data (Hopley et al. 2021). We used target capture sequencing (Angiosperms353 bait kit) and performed phylogenetic analyses using the workflow described in Chen et al. 2024 to better understand the evolutionary relationships between the target weed and its relatives. The sampling included all native Australian species, commercially important varieties, and *Passiflora* species introduced to Australia, as well as other taxa included in the host test list. Species in Salicaceae (*Casearia completa* and *C. nitida* ) and Violaceae ( *Viola odorata* and *Melicytus ramiflorus*) were used as outgroups. A maximum likelihood tree was constructed using IQ‐TREE v2.2.0.5 using concatenated data. The alignment contained 219,042 columns with 55,303 parsimony‐informative sites. The final tree included 34 species. For time calibration, the maximum age of the root was set to 91 MYA according to the reconstructed age of Malphigiales (Wikström et al. 2001), and we used a calibration point for *Passiflora* from fossil seed (Mai 1967) at 37 Mya with the maximum age set by the 95% confidence interval at 48.46 Mya from a study on the adaptive radiation of *Adenia* (Hearn 2006). The strict, correlated, and discrete clock models had the same log likelihood, with relaxed being marginally lower. Therefore, the simplest model (strict) was chosen.

To explore topological uncertainty, we subselected 100 of 1000 (Senecioneae) or 10,000 (*Passiflora*) ultrafast bootstrap samples from IQ‐TREE and time calibrated them using the same model as for the maximum likelihood tree. We then calculated degrees of separation, phylogenetic distances on the phylogram, and phylogenetic distances on the chronogram across the 100 bootstrap samples to obtain means and standard deviations.

**FIGURE 4:**
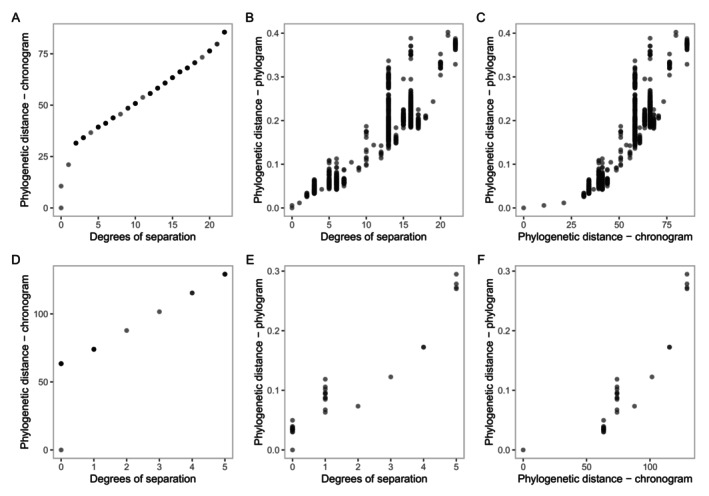
Measures of phylogenetic distance plotted against each other for a phylogenetic tree of Senecioneae with *Senecio vulgaris* as the hypothetical target weed (top row) and *Passiflora* with *P. foetida* as the target (bottom row). (A, D) phylogenetic distance on the chronogram increases monotonously with degrees of separation. (B, E) On the phylogram, phylogenetic distance varies for species with the same degree of separation. (C, F) On the chronogram, there are species with a higher phylogenetic distance when compared to the phylogenetic distance on the phylogram.

Phylogenetic distance on the chronogram increases monotonously with degrees of separation (and vice versa), meaning rank order is the same, but it does not increase in constant intervals (Figure 4A,D). Since the phylograms and chronograms within each dataset share the same topology (branching order), degrees of separation do not change between these phylogeny types, and this extends to if the phylogeny was turned into a cladogram. On the phylogram, phylogenetic distance from the target varies widely for members of the same clade due to varying rates of evolution across branches. Note that some species have a lower phylogenetic distance despite having a higher degree of separation from the target (Figure 4B,E). Therefore, and all else being equal, the implied order of the host test list would differ between using degrees of separation vs. phylogenetic distance when using these phylograms. Similarly, some species have a lower phylogenetic distance from the target on the phylogram despite higher phylogenetic distance on the chronogram (Figure 4C,F), that is, the clusters of points have shifted to the right compared to the previous two panels.

**FIGURE 5:**
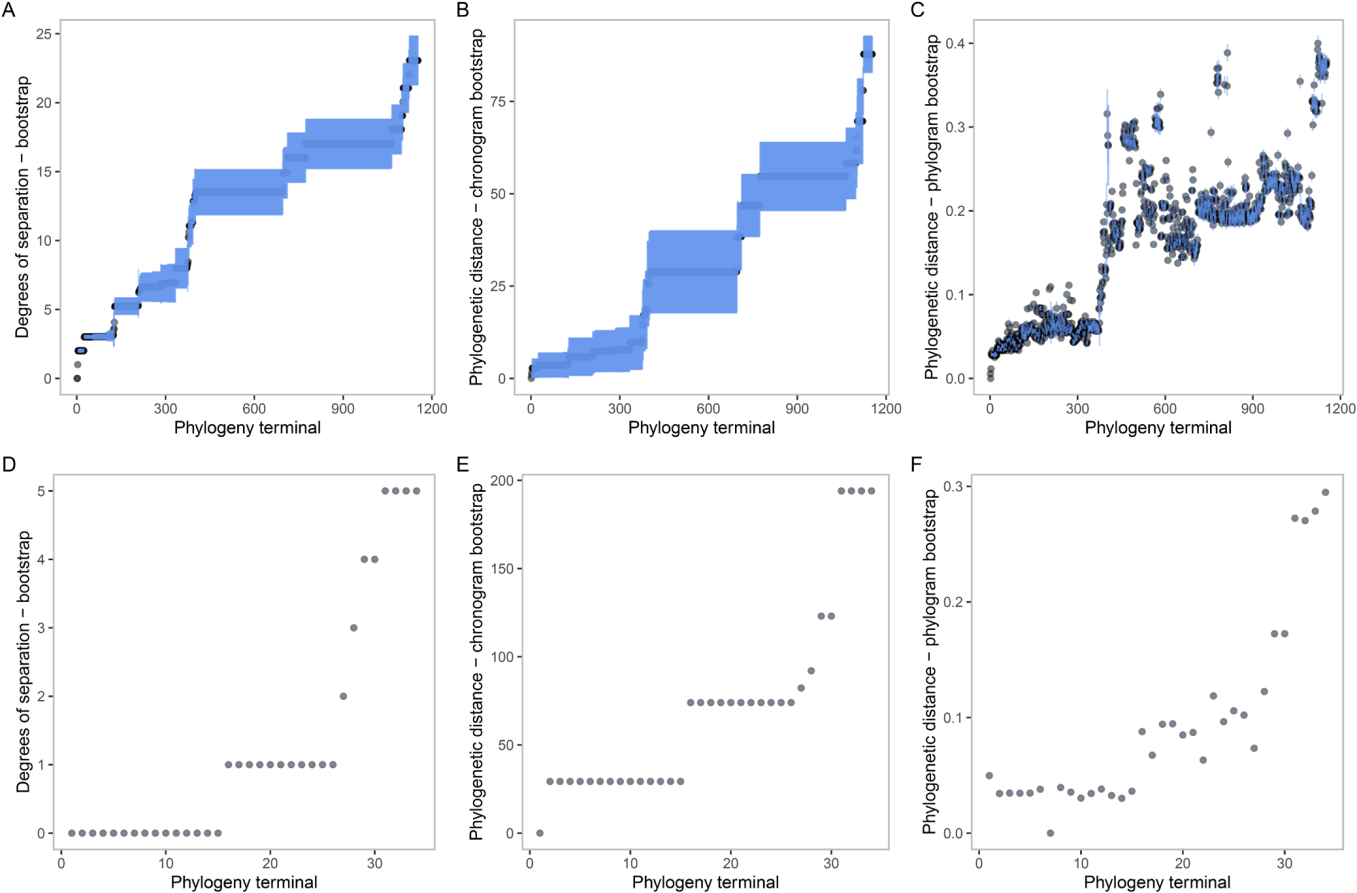
Uncertainty in measures of phylogenetic distance for a phylogenetic tree is higher in a Senecioneae Sanger data (top row) compared to a *Passiflora* target capture sequence data (bottom row). Points are means with uncertainty visualised as standard deviations (i.e., error bars in blue) across 100 ultrafast bootstrap samples with the X axis representing tree terminals sorted in ascending order of mean degrees of separation. (A, D) Degrees of separation. (B, E) Phylogenetic distance on chronogram. (C, F) Phylogenetic distance on phylogram.

The *Passiflora* dataset provides better phylogenetic resolution compared to the Senecioneae dataset due to the type of sequencing used; there is less topological uncertainty with error bars being markedly diminished (Figure 5). For *Passiflora*, the standard deviations for degrees of separation for all bootstrapped phylograms and chronograms are 0 (Figure 5D). Where there is more phylogenetic certainty, like in the Senecioneae phylogeny, the differences in degrees of separation and phylogenetic distance between some non‐target lineages are not meaningful once the error is considered. However, it is worth noting that depending on the availability of GenBank data, a relatively comprehensively sampled phylogeny using a handful of Sanger markers could be produced quickly and without sequencing costs and still provide some useful information about the relationship of a target weed to its relatives. Here, most of the Senecioneae data was taken from GenBank, with supplementary sequencing done for 32 species (Schmidt‐Lebuhn et al. 2020).

## 8 Conclusions and Future Directions

Different measures of relatedness can inform the design of host specificity test lists under the centrifugal phylogenetic method and its modernisations. Tools such as PhyloControl (Chen, Stevens, et al. 2025) can facilitate this process by enabling the visualisation of phylogenies and calculation of measures of relatedness. Furthermore, phylogenetic (patristic) distance offers distinct advantages over more simplistic measures like degrees of separation. By incorporating both tree topology and branch length information, phylogenetic distance allows for better prioritisation of species for biocontrol risk analysis. It also provides a better reflection of phylogenetic uncertainty, as poorly supported branches are often short and therefore contribute minimally to the score. For the same reason, phylogenetic uncertainty is smaller for phylogenetic distance than for degrees of separation across a near‐optimal sample of tree space.

It is important, however, to consider the various types of branch lengths that can be found in phylogenies. Branch lengths on phylograms may better reflect divergence, but only if the practitioner is prepared to argue that divergence in the data underlying the phylogenetic tree is correlated with divergence in the relevant ecological, biochemical, and physiological traits that would affect host specificity. Branch lengths on chronograms reflect relatedness as commonly understood, but they do not account for shifts in the rate of evolution and may thus over‐ or underestimate the divergence in important traits between two species.

Degrees of separation may be applied to any published phylogenetic tree, even if the underlying data are unavailable. However, leveraging branch length information requires access to the phylogenetic trees and ideally the underlying data matrices. This provides an impetus for making phylogenetic data available in publicly accessible data portals. Improving data accessibility and transparency will support more rigorous and reproducible applications of phylogenomics in the design of host test lists for biological control.

## Disclosure

*Benefit sharing statement*: All raw sequencing data have been shared with the broader public via appropriate biological databases.

## Conflicts of Interest

The authors declare no conflicts of interest.

## Data Availability

The phylogenies and sequence alignments used in the case study are available on the CSIRO Data Access Portal (https://doi.org/10.25919/xvwt‐vv39). The *Passiflora* raw target capture sequencing data are available on the NCBI Sequence Read Archive (SRA) under BioProject PRJNA1230843.
